# Structural Recognition of Triple-Stranded DNA by Surface-Enhanced Raman Spectroscopy

**DOI:** 10.3390/nano11020326

**Published:** 2021-01-27

**Authors:** Luca Guerrini, Ramon A. Alvarez-Puebla

**Affiliations:** 1 Department of Physical and Inorganic Chemistry—EMaS, Universitat Rovira I Virgili, Carrer de Marcel∙lí Domingo s/n, 43007 Tarragona, Spain; 2 ICREA, Passeig Lluís Companys 23, 08010 Barcelona, Spain

**Keywords:** surface-enhanced Raman spectroscopy, DNA, triplex, plasmonic nanoparticles

## Abstract

Direct, label-free analysis of nucleic acids via surface-enhanced Raman spectroscopy (SERS) has been continuously expanding its range of applications as an intriguing and powerful analytical tool for the structural characterization of diverse DNA structures. Still, interrogation of nucleic acid tertiary structures beyond the canonical double helix often remains challenging. In this work, we report for the first time the structural identification of DNA triplex structures. This class of nucleic acids has been attracting great interest because of their intriguing biological functions and pharmacological potential in gene therapy, and the ability for precisely engineering DNA-based functional nanomaterials. Herein, structural discrimination of the triplex structure against its duplex and tertiary strand counterparts is univocally revealed by recognizing key markers bands in the intrinsic SERS fingerprint. These vibrational features are informative of the base stacking, Hoogsteen hydrogen bonding and sugar–phosphate backbone reorganization associated with the triple helix formation. This work expands the applicability of direct SERS to nucleic acids analysis, with potential impact on fields such as sensing, biology and drug design.

## 1. Introduction

The polymorphic nature of DNA enables the adoption of a diverse range of structural arrangements beyond the right-handed B-form duplex, including non-canonical structures such as G-quadruplexes, triplexes, and i-motifs [1]. These structures have been shown to participate in multiple biological processes and, remarkably, their forming sequences have been recognized as sources of genetic instability, contributing to human diseases and genetic disorders [1,2].

Among non-canonical conformations, triplex structures have attracted great interest. Besides the understanding of their biological functions, they display pharmacological potential in gene therapy based on their ability to modulate gene expression, such as via inhibition of disease-related genes and generation of site-specific mutations [2,3]. Additionally, triplex nucleic acids have been emerging as a powerful tool in the field of DNA nanotechnology where their ability for structural reconfiguration is exploited for engineering DNA-based functional nanomaterials via a diverse set of external stimuli [4,5].

Structurally speaking, triplex nucleic acids can be classified as intramolecular structures that are naturally occurring at DNA regions containing homopurine and homopyrimidine segments with mirror repeat symmetry, or intermolecular multistrand assemblies resulting from the binding of a triplex-forming oligonucleotide (TFO) to a target duplex structure [1,3]. TFOs can bind double helices with high affinity and sequence specificity via Hoogsteen hydrogen bonds, positioning in the major groove of the duplex. These triplex-forming strands consist of pyrimidine or purine-rich sequences that bind their counterparts (a purine or pyrimidine segment) in the double helix in either parallel or anti-parallel direction [2,3]. Besides the nucleobase sequence and length, surrounding conditions also play a central role in the generation of stable triplex assemblies. These variables include the solution pH and the presence of divalent cations (e.g., Mg^2+^) or naturally occurring polyamines (e.g., spermine), which can act as triplex stabilizers by reducing the extent of electrostatic repulsion between the phosphate backbones [2,3,4].

In-depth characterization of the structural, kinetic and thermodynamic properties of triplexes is of utmost importance for the correct understanding of their biological role and to improve their efficiency and specificity in gene therapy [3,4]. Techniques, such as nuclear magnetic resonance (NMR), X-ray fiber diffraction (XRD), and other optical spectroscopies (e.g., circular dichroism, UV-Vis and fluorescence-based methods) have been preferably applied to this purpose [4,6]. To a much lesser extent, structural data on triplex structures have been also collected using Raman spectroscopy [7,8,9,10]. Raman spectroscopy is a vibrational technique which has been lengthily exploited for the detailed structural characterization of biomolecules, including nucleic acid structures. Importantly, the possibility of performing Raman studies on nucleic acids in their natural state (i.e., in solution, rather than in crystal form such as for XRD) increases the biological significance of the obtained data. However, the Raman scattering phenomenon is a very inefficient process, which drastically limits the sensitivity of this spectroscopy and, thus, hampers its application to molecular systems at low-concentration levels. This issue can be overcome via plasmon-mediated enhancement of the Raman scattering from a molecular entity located near a metallic nanostructured surface when localized surface plasmon resonances (LSPRs) are efficiently excited upon illumination with an appropriate light source [11]. The integration of Raman technique and plasmonics paved the way for the development of a new method termed surface-enhanced Raman spectroscopy (SERS) which preserves the structural specificity, high experimental flexibility and simplicity of the traditional method while imparting extremely high sensitivity. This enabled the application of SERS as an ultrasensitive analytical approach for the structural characterization of (bio)molecular species as well as sensing tool for their detection and quantification [11,12,13]. However, while the use of SERS in indirect approaches for nucleic acids sensing has been extensively dealt with in the literature, its wider use as an ultrasensitive analytical tool for this class of biomolecules via the acquisition of their intrinsic SERS vibrational fingerprinting (i.e., direct SERS) has been traditionally far less explored, until relatively recently [14,15,16,17,18,19,20,21,22]. Our group devised a novel approach based on the use of positively charged silver colloids as plasmonic-enhancers which successfully tackled prior issues associated with the reproducible acquisition of reliable SERS spectra of nucleic acids at low concentrations [19,23,24]. Notably, the use of this class of silver nanoparticles affords high sensitivity for discriminating DNA conformations (i.e., single- vs. double-stranded helix) which is otherwise poor for traditional negatively charged colloids [25].

To the best of our knowledge, only two articles from 1996 reported the acquisition of triplex SERS spectra, specifically from RNA triple helices adsorbed on silver electrodes [26,27]. However, the illustrated SERS data display spectral profiles with no actual resemblance to the characteristic vibrational patterns of nucleic acids. This incongruence was later elucidated by noting the striking correspondence of these anomalous spectra to the SERS of methylviologen, a molecule commonly used in electrochemistry such as for electrode roughening, which has been indicated as the actual source of the intense signal [28].

Herein, we report thus the first unequivocal recognition of a DNA triplex structure by SERS spectroscopy. Specifically, we investigated an intermolecular triple-helix comprising a 31-base pair duplex bound to a 22-nucleotide pyrimidine-rich single strand. The molecular fingerprint information contained in the SERS spectra allowed for the structural discrimination and characterization of the free single strand and the same sequence involved in the triplex construct. This study expands the applicability of SERS spectroscopy as a powerful molecular tool for structural interrogation of nucleic acids to the intriguing class of triplex structures, with potential impact on fields such as sensing, biology, drug-design and DNA nanotechnology.

## 2. Materials and Methods

### 2.1. Materials

All chemicals were obtained from Sigma-Aldrich and Fisher Scientific at the highest purity available. Lyophilized oligonucleotides were purchased from Eurofins and dissolved in milli-Q water to approximately 300 μM concentration. The exact DNA concentrations were then estimated by measuring the absorbance at 260 nm. Subsequently, DNA samples were diluted to 40 μM in triplex buffer (sodium-magnesium phosphate buffer solution pH 7, 120 mM NaCl, 8 mM Mg^2+^). Duplex formation was achieved by heating to 90 °C for 10 min an equimolar solution (20 µM) of complementary strands in triplex buffer. The solution was then slowly cooled down to room temperature. All DNA solutions were kept at 4 °C before their experimental use. Triplex formation was performed by adding an equimolar amount of the triplex-forming oligonucleotide (TFO) to the duplex nucleic acid in triplex buffer. The mixture was allowed to equilibrate at 4 °C for 24 h.

### 2.2. Synthesis of Positively Charged Silver Colloids and SERS Measurements

Synthesis of positively charged spermine coated-silver nanoparticles (AgSp) was carried out as previously reported [29,30]. Briefly, to 10 mL of Milli-Q water were consecutively added, under stirring, 20 μL of silver nitrate 0.5 M and 7 μL of spermine tetrahydrochloride 0.1 M solutions. After 20 min, 250 μL of sodium borohydride 0.01 M were quickly added to the mixture under vigorous stirring. The colloids were then left to settle overnight and the deposit at the bottom of the vial was discarded. To prevent the adhesion of AgSp nanoparticles onto glass surfaces, vials were previously coated with polyethyleneimine. Samples for SERS experiments were prepared as follows. All DNA samples were previously diluted to 20 µM with their corresponding solvent (milli-Q water or triplex buffer). Then, 0.9 µL of these solutions were added to 150 µL of colloids (stored at 4 °C), and quickly stirred. Samples were left to equilibrate for at least 3 h at 4 °C, and briefly sonicated before the acquisition of the SERS spectra.

### 2.3. Instrumentation

Scanning UV-vis spectra were obtained using a Thermo Scientific Evolution 201 UV-visible spectrophotometer. Transmission electron microscopy, TEM, images were collected using a JEOL 1011 (JEOL USA, Inc, Peabody, MA) operating at 100 kV. TEM samples were prepared by diluting the sample before their deposition onto carbon−Formvar-coated 200 mesh copper grids. SERS spectra were obtained using a Renishaw InVia Reflex confocal microscope equipped with a 514 nm laser. A lens for macrosampling (30 mm focal length, 0.17 NA) was employed to focus the laser onto the sample. The averaged SERS spectra illustrated in the manuscript were typically obtained under the following conditions: 10 s exposure time, 15 accumulations, 100% laser power.

## 3. Results and Discussion

**Figure 1:**
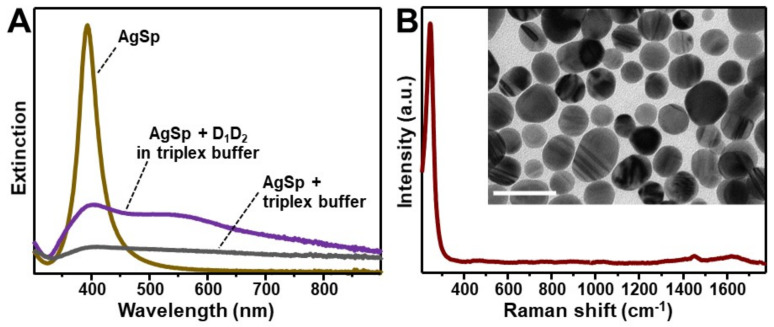
(**A**) Extinction spectrum of spermine-coated silver colloids (AgSp, 150 μL) before and 3h after the addition of 0.9 μL of (a) triplex buffer (grey line) or (b) a 20 μM solution of D_1_D_2_ duplex in triplex buffer (purple line). (**B**) Surface-enhanced Raman spectroscopy (SERS) spectrum of AgSp upon addition of 0.9 µL of triplex buffer. The intense band at 244 cm^−1^ is ascribed to the ν(Ag–Cl) vibration. Inset: representative transmission electron microscopy (TEM) image of the nanoparticles (scale bar: 50 nm).

The plasmonic substrate for SERS characterization of nucleic acids consists of colloidal spermine-coated silver nanoparticles (AgSp) with a narrow LSPR centered at ca. 390 nm (Figure 1A) and an average diameter of 20.0 ± 4.1 nm (Figure 1B, inset and Figure S1A). Polyaminic spermine molecules adsorbed onto the silver surface impart an overall positive charge to the silver nanostructures (ζ-potential = +40 mV). The addition of tiny amounts of triplex buffer solution (sodium-magnesium phosphate buffer solution pH 7, 120 mM NaCl, 8 mM Mg^2+^) induces a relatively slow but unrestrained nanoparticle aggregation (Figure 1A). On the other hand, the resulting SERS spectrum (Figure 1B) shows a low background signal in the spectral region of interest for DNA analysis (ca. 400–1800 cm^−1^).

**Figure 2:**
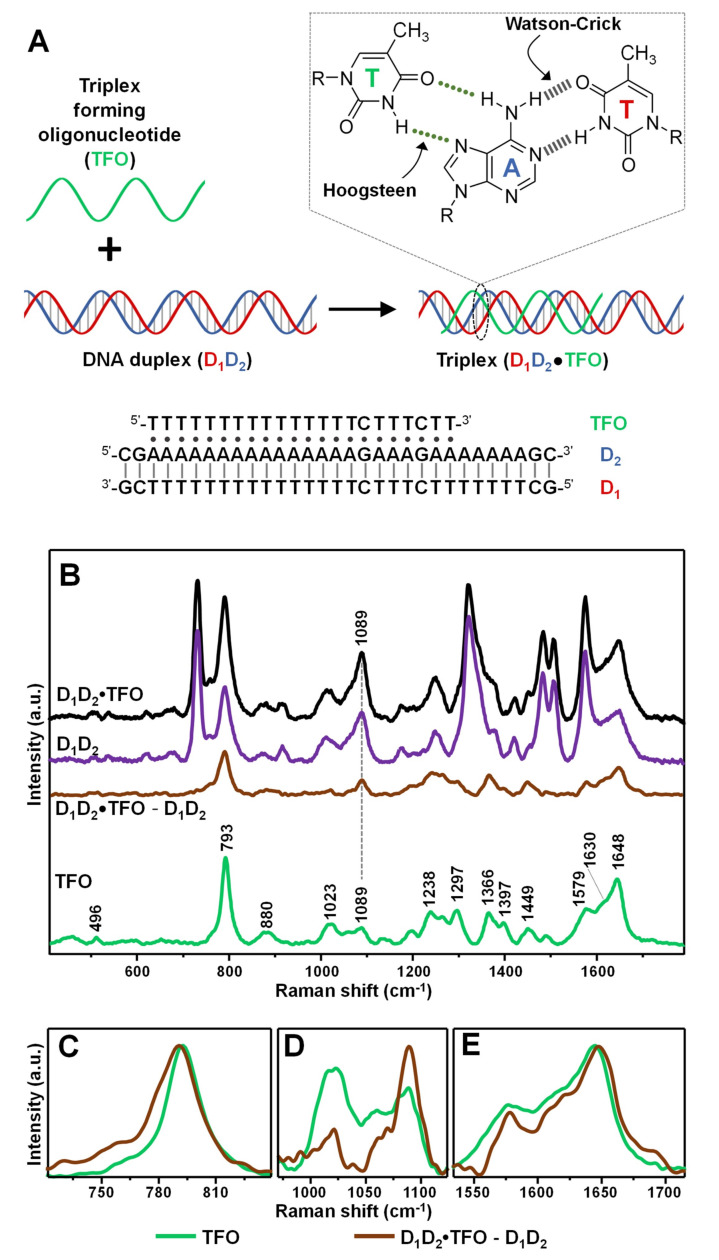
(**A**) Outline of the intermolecular triplex formation and corresponding oligonucleotide sequences (where “•” and “-” indicates Hoogsteen and Watson–Crick base pairings, respectively). Inset: chemical structure of a parallel T•AT triplet. (**B**) SERS spectra of the intermolecular triplex D_1_D_2_•TFO, duplex D_1_D_2_, and the individual TFO. The D_1_D_2_•TFO and D_1_D_2_ SERS spectra were normalized to the intensity of the νPO_2_^-^ band at 1089 cm^−1^ according to the relative content of phosphate groups in the construct (i.e., 84 for D_1_D_2_•TFO, and 62 for D_1_D_2_). D_1_D_2_•TFO—TFO is the difference SERS spectrum obtained upon digital subtraction of the duplex spectrum to the triplex spectrum. (**C**–**E**) Details of the 720–840, 960–1180 and 1530–1715 cm^−1^ spectral regions, respectively, for TFO spectrum (green curve) and difference D_1_D_2_•TFO—TFO spectrum (brown curve). For the sake of a better comparison, the intensities of the spectra were arbitrarily modified in each of these three figures.

As a model intermolecular triple-helix (Figure 2A), we selected one based on the association of a 31-base pair duplex (D_1_D_2_), comprising a pyrimidine-rich strand (D_1_) and a purine-rich strand (D_2_), with a 22-nucleotide pyrimidine-rich strand (TFO). The third strand binds via formation of 19 T•AT and 2 C•GC base triplets adopting a parallel orientation along the major groove of the purine-rich D_2_ strand in the Watson–Crick D_1_D_2_ duplex [31]. The resulting D_1_D_2_•TFO construct (1:1:1 ratio, where “•” indicates the Hoogsteen interactions) has been previously characterized by conventional techniques, demonstrating to possess high stability at room temperature in a pseudo-physiological triplex buffer (pH 7, 120 mM NaCl, 8 mM Mg^2+^) [31]. The triplex construct was obtained by combining an equimolar amount of TFO and D_1_D_2_ duplex for 24 h at 4 °C. In fact, while generating very stable structures, triplex formation is kinetically much slower than duplex annealing [2].

Identical volumes of DNA containing triplex buffers of the different samples (i.e., duplex D_1_D_2_, triplex D_1_D_2_•TFO, and single-strand TFO) were added to AgSp colloids. Differently to what previously observed (Figure 1A), the nanoparticles undergo rapid aggregation into stable clusters in suspension due to the electrostatic adhesion of the nucleic acids mediated by the phosphate backbone-spermine interactions. Representative transmission electron microscopy (TEM) images of the AgSp colloids upon addition of either triplex buffer or D_1_D_2_ in triplex buffer (Figure S1B,C) provide complementary qualitative evidence of the different degree of aggregation underwent by the nanoparticles.

Figure 2B shows the corresponding SERS spectra. Typically, difference spectroscopy is applied to both Raman and SERS analysis of nucleic acids to better expose the spectral differences emerging from the complex vibrational patterns of these large biomolecules. To this end, digital subtraction between spectra is carried out using the νPO_2_^-^ band at ca. 1089 cm^−1^ as internal standard, due to its large insensitivity to structural alterations [14]. In our specific case, since duplex and triplex structures possess different molar content of phosphate groups (i.e., 84/mol for D_1_D_2_•TFO, and 62/mol for D_1_D_2_), the intensity of their corresponding SERS spectra was proportionally adjusted to the peak height of the ca. 1089 cm^−1^ band according to the relative number of PO_2_^-^ per molecular construct. This allows extracting a difference spectrum D_1_D_2_•TFO − D_1_D_2_ which primarily represents the vibrational pattern of TFO in the triplex structures and, thus, can be meaningfully compared to the SERS profile of the unbound TFO strand. The SERS spectrum of TFO is dominated by thymine bands [25], as expected from the relative nucleobase content. Still, it can be distinguished a shoulder approximately located at 1630 cm^−1^ which has been attributed ascribed to ν(C_2_=O_4_) modes of cytosine. Additionally, based on our previous works [23,25,29], we assume non-negligible contributions from cytosine in-plane ring vibrations in the complex ensemble of overlapping features in the 1150–1600 cm^−1^ range as well as for the ring breathing band at ca. 792 cm^−1^. Figure 2C–E illustrate the SERS signals of TFO and the digitally subtracted D_1_D_2_•TFO − D_1_D_2_ in three key spectral regions of interest. For the sake of comparison, the intensities of the two spectra were arbitrarily modified to better visualize the discussed spectral changes. The intense feature centered at ca. 792 cm^−1^ of TFO (Figure 2C, green curve) undergoes a 3 cm^−1^ blue-shift and a broadening in the D_1_D_2_•TFO − D_1_D_2_ difference spectrum (Figure 2C, brown curve), which is informative of a perturbation of the nucleobase electronic properties associated with an increase in base stacking. The extent of spectral shift is significantly lower than the one observed upon hybridization of a polythymine strand with a complementary polyadenine sequence to form the corresponding duplex (ca. 7 cm^−1^ blue-shift) [29], which is consistent with the knowledge that the stacking interactions of TFO nucleobases in triplex constructs are weaker [1]. This spectral change is mirrored by the ca. 4 cm^−1^ red-shift of the thymine ν(C_4_=O_4_) band at 1644 cm^−1^ (Figure 2E), primarily sensitive to base pairing, which suggests the involvement of the carbonyl group in hydrogen bonding (Figure 2A, inset) [25]. Finally, clues of changes in the sugar-phosphate backbone conformation are mostly reflected in the 800–1100 cm^−1^ spectral range. Here, we observe the clear change in intensity ratio between the vibration of the ribose sugar at ca. 1020 cm^−1^ and the abovementioned νPO_2_^−^ band at ca. 1089 cm^−1^. A similar trend was also previously reported for polythymine strands forming double helix structures [29,32]. The set of all spectral changes is therefore consistent with the binding of the TFO to the purine-rich strand in the D_1_D_2_ duplex via Hoogsteen base pairing and stacking interactions with the neighboring bases. It is also worth noting that, while not vibrationally interpreted due to the intricacy of band overlapping, relevant spectral alterations were also detected in the 1200–1450 cm^−1^ spectral range (Figure S2). The SERS spectra in Figure 2B are the averaged results of three separate replicas, whose original non-baseline spectra are illustrated in Figure S3 (the data show high reproducible spectral profiles with only minimal fluctuations of the broad continuum background).

**Figure 3:**
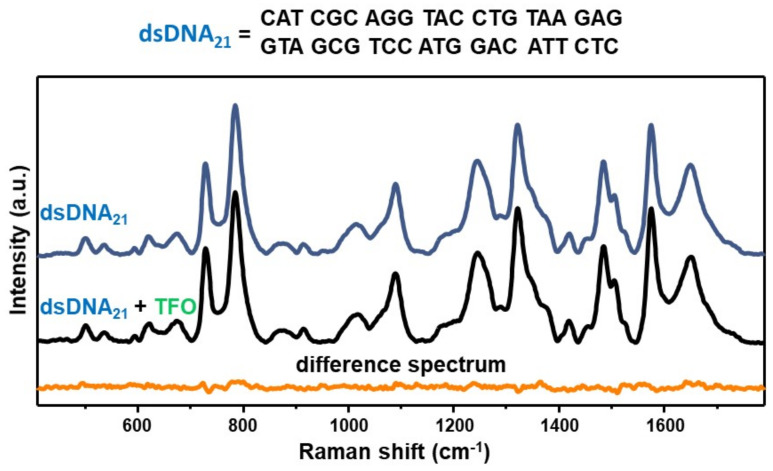
SERS spectra of the 21-base pairs duplex dsDNA_21_ (blue curve) and the equimolar mixture dsDNA_21_ + TFO (black curve). Orange curve: difference spectrum (dsDNA_21_ + TFO)—dsDNA_21_ obtained by digitally subtracting the blue curve to the black one.

As an experimental control, we acquired the SERS spectrum of an equimolar mixture of the TFO and a non-complementary 21-bp duplex (dsDNA_21_) and compared to the SERS signal of the duplex alone via digital subtraction (Figure 3), as similarly performed for D_1_D_2_ in Figure 2B. In this case, the corresponding difference spectrum (dsDNA_21_ + TFO)—dsDNA_21_ does not reveal any distinguishable vibrational pattern. This result is not surprising considering our previous SERS study on mixtures of a 21-nt single-stranded DNA (ssDNA) and a 21-bp duplex, which showed that the SERS signal is fully dominated by the duplex contribution for single-strand content below 80% [29]. Such outcome was attributed to a superior ability of DNA duplexes in mediating the formation of highly efficient interparticle hotspots as compared to single-stranded sequences, which may be based on the different availability of negatively charged phosphate groups. Thus, the experimental control further corroborates the validity of the data presented in Figure 2, that is the acquired SERS spectrum of the D_1_D_2_ + TFO equimolar mixture indeed represents the vibrational fingerprint of the intermolecular triplex.

In summary, structural discrimination of the triplex structure against its duplex and tertiary strand counterparts is unambiguously revealed by recognizing key markers bands in their intrinsic SERS fingerprints. These vibrational features are informative of the base stacking, Hoogsteen hydrogen bonding and sugar–phosphate backbone reorganization associated with the triple helix formation. This work paves the way for the application of direct, label-free analysis of nucleic acids via SERS spectroscopy beyond the single strand DNAs and canonical double helix forms to this intriguing class of tertiary structures which captivated the attention due to, among others, their biological functions and pharmacological potential in gene therapy.

## Data Availability

The data presented in this study are available on request from the corresponding authors.

## Supplementary Materials

The following are available online at https://www.mdpi.com/2079-4991/11/2/326/s1, Figure S1: size histogram of the silver nanoparticles, TEM images of the colloids upon addition of triplex buffer or D_1_D_2_ in triplex buffer. Figure S2: SERS spectrum of TFO and difference (D_1_D_2_•TFO—D_1_D_2_) spectrum in the 450–1750 cm^−1^ range. Figure S3: SERS spectra of TFO, D_1_D_2_•TFO and D_1_D_2_ from different replicas.
