# Supplementary material for: Structural Recognition of Triple-Stranded DNA by Surface-Enhanced Raman Spectroscopy
Source: Nanomaterials (Basel). 2021 Jan 27;11(2):326. doi: 10.3390/nano11020326 (PMC7912272; doi:10.3390/nano11020326)
Supplement: Supplementary file 1 [file nanomaterials-11-00326-s001.pdf]

SUPPLEMENTARY INFORMATION

Structural Recognition of Triple-Stranded DNA by Surface-Enhanced Raman Spectroscopy

Luca Guerrini [1,*] and Ramon A. Alvarez-Puebla [1,2,*]

[1] Department of Physical and Inorganic Chemistry—EMaS, Universitat Rovira I Virgili, Carrer de Marcel·lí Domingo s/n, 43007 Tarragona, Spain
[2] ICREA, Passeig Lluís Companys 23, 08010 Barcelona, Spain.
* Correspondence: luca.guerrini@urv.cat (L.G.); ramon.alvarez@urv.cat (R.A.A.-P.)

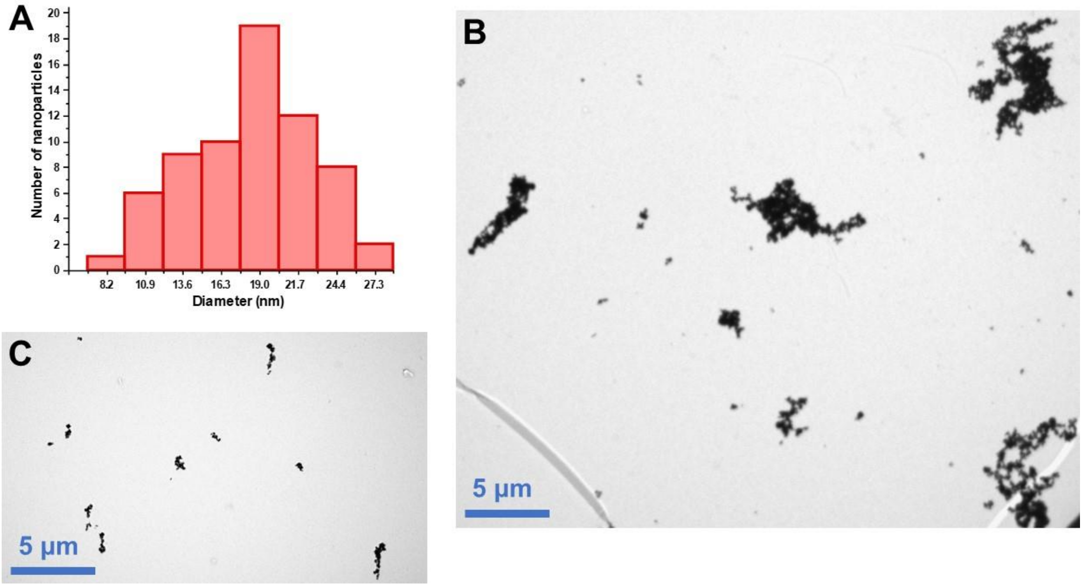

Figure S1. (A) Size histograms of AgSp nanoparticles (N = 70). (B, C) Representative TEM image of AgSp colloids (150 µL) upon addition of 0.9 µL of (B) triplex buffer or (C) a 20 µM solution of D_1D_2 duplex in triplex buffer. Triplex buffer: sodium-magnesium phosphate buffer solution pH 7, 120 mM NaCl, 8 mM Mg^{2+}.

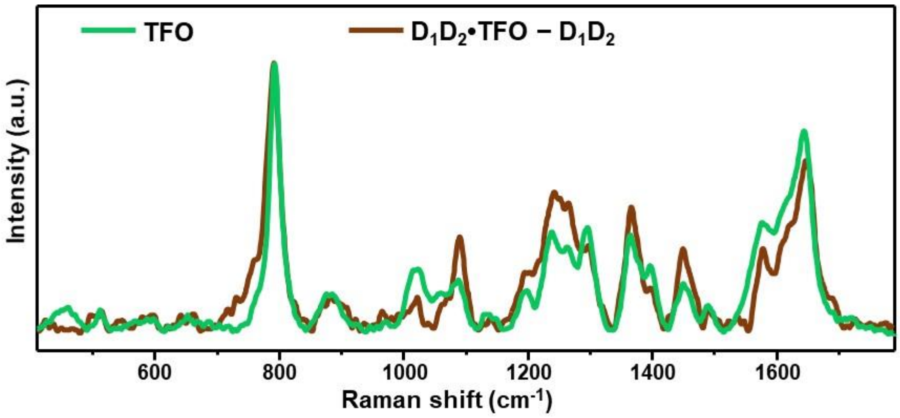

Figure S2. SERS spectrum of TFO and difference spectrum D_1D_2•TFO – D_1D_2. For the sake of a better comparison, the intensity of the spectra was arbitrarily normalized to the intensity of the ring breathing band at ca. 790 cm^{-1}.

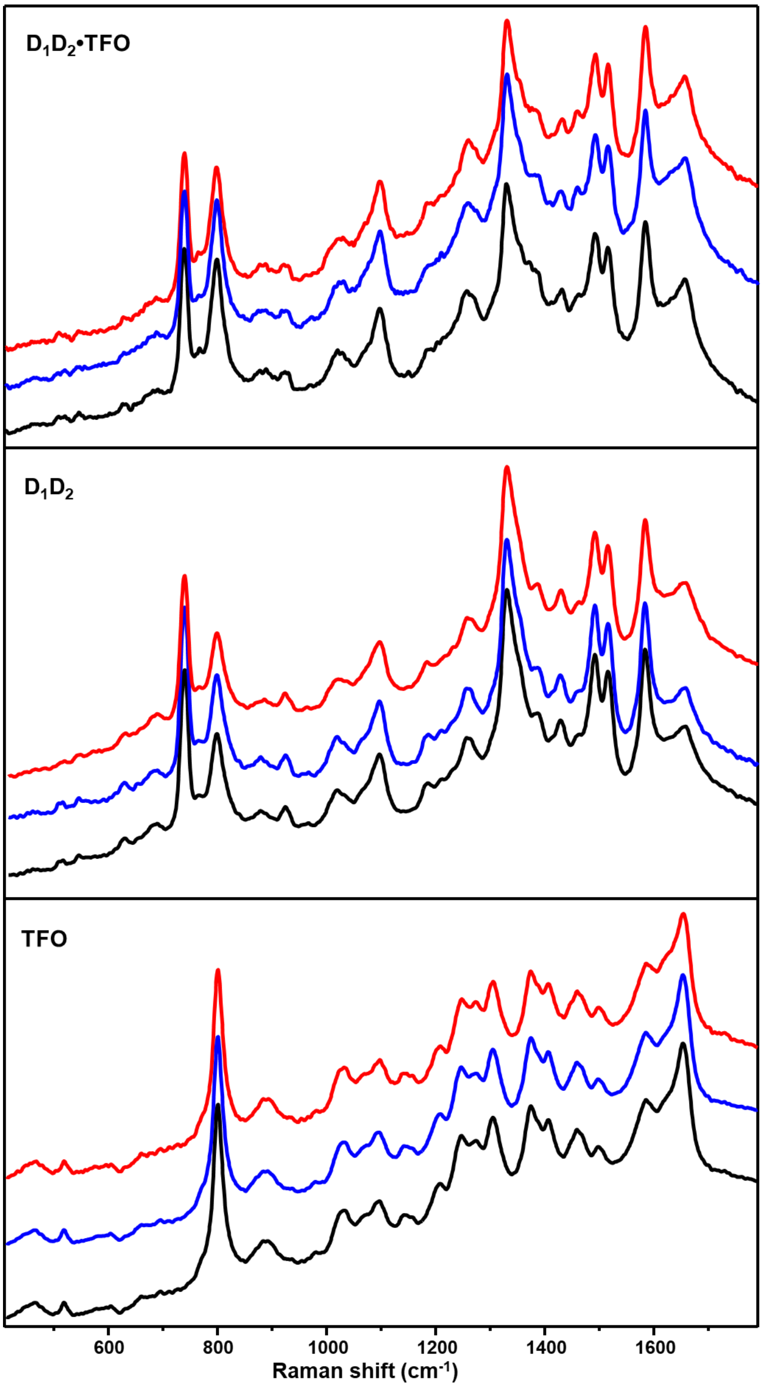

Figure S3. Original non-baseline SERS spectra of TFO, D_1D_2•TFO and D_1D_2 from different replicas.
